# Risk factors associated with malnutrition in elderly patients with chronic heart failure

**DOI:** 10.1097/MD.0000000000049680

**Published:** 2026-07-17

**Authors:** Jianfeng Long, Limin Deng, Jiandong Zhang, Shiping Liu, Kang Zhou

**Affiliations:** a Department of Clinical Nutrition, The Second Xiangya Hospital of Central South University, Changsha, Hunan Province, China; b Department of Internal Medicine,Yantai Fushun Hospital, Yantai, Shandong Province, China; c Department of Cardiovascular Surgery, The Second Xiangya Hospital of Central South University, Changsha, Hunan Province, China.

**Keywords:** C-reactive protein, chronic heart failure, GLIM criteria, malnutrition, New York Heart Association, NT-proBNP

## Abstract

This retrospective observational study aimed to identify clinical determinants of malnutrition in elderly patients with chronic heart failure. Elderly patients (≥65 years) with established chronic heart failure managed at a single tertiary hospital between January 2022 and December 2024 were included. Malnutrition was diagnosed according to the Global Leadership Initiative on Malnutrition criteria, using a 2-step approach combining risk screening and phenotypic and etiologic assessment. Demographic, anthropometric, comorbidity, laboratory, and heart failure-related data were extracted from electronic medical records. Univariate and multivariate logistic regression analyses were performed to explore factors associated with malnutrition, and model performance was evaluated using receiver operating characteristic analysis. Among 203 patients, 36 (17.7%) were classified as malnourished. Compared with non-malnourished patients, those with malnutrition were older and had a lower body mass index, lower serum albumin and prealbumin, higher C-reactive protein and neutrophil-to-lymphocyte ratio, poorer renal function, higher N-terminal pro-B-type natriuretic peptide levels, and a higher prevalence of New York Heart Association class III/IV and chronic obstructive pulmonary disease. In multivariate analysis, older age, lower body mass index, higher New York Heart Association class, elevated C-reactive protein, presence of chronic obstructive pulmonary disease, reduced estimated glomerular filtration rate, and higher N-terminal pro-B-type natriuretic peptide remained independently associated with malnutrition. The final model showed good discriminatory performance, with an area under the curve of 0.902, supporting its potential utility for nutritional risk stratification in this population.

## 1. Introduction

Chronic heart failure (CHF) is a major cause of morbidity and mortality in older adults and is frequently accompanied by functional decline, frailty, and complex multimorbidity. With population aging and improved survival after acute cardiovascular events, the number of elderly individuals living with CHF continues to increase, making the optimization of global clinical management, including nutritional care, an important priority in cardiology and geriatric practice.^[1,2]^ Malnutrition is highly prevalent in patients with CHF and is associated with impaired quality of life, longer hospital stays, higher readmission rates, and increased mortality. Recent outpatient cohorts have reported that more than half of patients with stable heart failure meet diagnostic criteria for malnutrition according to the global leadership initiative on malnutrition (GLIM), and many have concomitant sarcopenia and functional dependence.^[3]^ Meta-analytic data further suggest that GLIM-defined malnutrition is consistently associated with increased short- and long-term mortality across diverse clinical populations.^[4]^ In older heart failure populations, nutritional risk scores such as the Controlling Nutritional Status score and related indices have demonstrated prognostic value for all-cause mortality and rehospitalization, underscoring the clinical relevance of systematic nutritional assessment in this group.^[5]^

The mechanisms linking CHF and malnutrition are multifactorial. Chronic neurohormonal activation, systemic low-grade inflammation, catabolic–anabolic imbalance promote muscle wasting and weight loss, while gut edema, early satiety, and reduced intestinal perfusion impair nutrient intake and absorption. Inflammatory and nutrition-related biomarkers, such as C-reactive protein (CRP), albumin-based indices, and composite scores integrating inflammatory and nutritional parameters, have been shown to correlate with prognosis in heart failure and to reflect the interaction between inflammation, nutritional status, and immune function.^[6]^ In parallel, the GLIM framework has provided standardized diagnostic criteria that combine phenotypic features (weight loss, low body mass index [BMI], and reduced muscle mass) with etiologic factors (reduced intake or assimilation and disease-related inflammation) for the diagnosis and grading of malnutrition in adults.^[7]^ Despite growing evidence on the prognostic impact of malnutrition in HF, several gaps remain. Many studies have focused on mixed-age or acute heart failure cohorts, and relatively few have specifically addressed elderly patients with chronic, stable CHF, in whom age-related physiological changes, renal dysfunction, pulmonary comorbidities, and polypharmacy may further modify nutritional risk. Furthermore, although some recent work has proposed risk models or nomograms for malnutrition in CHF, these often incorporate specialized measures that may not be routinely available in all settings.^[8]^ There is a need to clarify which readily obtainable demographic, clinical, and laboratory parameters, such as BMI, heart failure severity indices, inflammatory markers, renal function indices, and major comorbidities, are most strongly associated with malnutrition in elderly CHF, and whether they can be combined into a pragmatic prediction model suitable for use in routine clinical practice.

In this context, the present study focuses on elderly patients with CHF and aims to characterize the baseline clinical and biochemical profile associated with malnutrition, identify independent risk factors using multivariable analysis, and evaluate the discriminative performance of a model constructed from variables that are widely available in daily care.

## 2. Methods

### 2.1. Study design

This study was approved by the Ethics Committee of The Second Xiangya Hospital of Central South University. This retrospective observational study included elderly patients diagnosed with CHF who received inpatient or outpatient management at our institution between January 2022 and December 2024. Eligible participants were identified through the electronic medical record system. Inclusion criteria were: age ≥65 years; confirmed diagnosis of CHF based on clinical presentation, echocardiographic findings, and guideline-based diagnostic criteria; and availability of complete demographic, clinical, laboratory, and nutritional assessment data. Exclusion criteria were: acute decompensated heart failure at the time of evaluation; severe concomitant diseases that could independently influence nutritional status, such as advanced malignancy, severe hepatic or renal dysfunction, or active systemic infection; history of major gastrointestinal disorders causing malabsorption; prior implantation of mechanical circulatory support devices; and incomplete or missing key clinical variables. The study was conducted in accordance with the ethical principles outlined in the Declaration of Helsinki and was approved by the institutional medical ethics committee. Written informed consent was obtained from all participants or their legally authorized representatives prior to data collection.

### 2.2. Diagnostic criteria for malnutrition

Malnutrition was diagnosed according to the consensus criteria proposed by the GLIM. The diagnostic process followed a 2-step approach, beginning with risk screening using a validated tool, followed by diagnostic assessment for individuals identified as being at nutritional risk. A diagnosis of malnutrition required the presence of at least 1 phenotypic criterion and 1 etiologic criterion.

Phenotypic criteria included: unintentional weight loss >5% within 6 months or >10% beyond 6 months; low BMI, defined as <20 kg/m^2^ for individuals aged 65 to 69 years and <22 kg/m^2^ for those aged ≥70 years; reduced muscle mass, assessed using bioelectrical impedance analysis, calf circumference, or other validated muscle mass indices.

Etiologic criteria included: reduced food intake or assimilation, characterized by an energy intake <50% of estimated requirements for >1 week or <75% for >2 weeks, or the presence of gastrointestinal conditions causing malabsorption; presence of disease-related inflammation, indicated by CHF status accompanied by clinical evidence of systemic inflammatory response or elevated inflammatory biomarkers.

Patients meeting both categories of criteria were classified as having malnutrition. These criteria were uniformly applied by trained clinicians to ensure diagnostic consistency.

### 2.3. Data collection

Clinical data were obtained retrospectively from the electronic medical records of elderly patients with CHF who met the eligibility criteria and were treated at the study institution between January 2022 and December 2024. For each patient, an index hospitalization or outpatient visit was defined as the time point at which nutritional status was assessed, and all baseline variables were extracted with reference to this encounter. Data extraction was performed using a standardized template by 2 trained investigators, and inconsistencies were resolved by consensus to reduce recording errors and missing information. Demographic and lifestyle information included age and sex, as well as current smoking and current alcohol use, which were derived from admission notes and structured medical history forms. Anthropometric variables comprised body weight and height, measured by nursing staff using calibrated equipment according to hospital protocols. BMI was calculated as weight divided by height squared (kg/m^2^). Where available, the results of routine nutritional screening were recorded using the Mini Nutritional Assessment-Short Form (MNA-SF).

Information on comorbid conditions was collected based on physician diagnoses documented in discharge summaries and longitudinal problem lists. The presence of hypertension, diabetes mellitus, chronic kidney disease, and chronic obstructive pulmonary disease (COPD) was recorded as binary variables. In addition, overall comorbidity burden was characterized descriptively in the cohort. Heart failure-related data were collected to reflect disease severity and functional status. New York Heart Association (NYHA) functional class at the index visit was determined by the responsible cardiologist according to established criteria. Biochemical markers of heart failure burden included plasma N-terminal pro-B-type natriuretic peptide (NT-proBNP), measured in the central laboratory using standard immunoassay techniques. Laboratory measurements were obtained from blood samples. These included hemoglobin, serum albumin, prealbumin, CRP, alanine aminotransferase (ALT), aspartate aminotransferase (AST), serum creatinine, and a complete blood count. The neutrophil-to-lymphocyte ratio (NLR) was calculated as the ratio of absolute neutrophil count to absolute lymphocyte count. Renal function was estimated using the Chronic Kidney Disease Epidemiology Collaboration formula to derive the estimated glomerular filtration rate (eGFR) (mL/min/1.73 m^2^) from serum creatinine, age, and sex. All biochemical and hematological parameters were processed in the hospital central laboratory according to internal quality control procedures.

### 2.4. Statistical analyses

Statistical analyses were performed using IBM SPSS Statistics, version 26.0 (IBM Corp.). Continuous variables were examined for distributional characteristics and are presented as mean ± standard deviation. Between-group comparisons of continuous variables (malnutrition vs non-malnutrition) were conducted using independent-samples *t* tests. Categorical variables are expressed as counts and percentages and were compared using the chi-square test or Fisher exact test, as appropriate. Univariate logistic regression analyses were performed to evaluate the association between each candidate predictor and the presence of malnutrition, and unadjusted odds ratios (ORs) with 95% confidence intervals (CIs) were calculated. Variables with *P* < .10 in univariate analyses were entered into a multivariate logistic regression model to identify independent predictors of malnutrition; adjusted ORs and 95% CIs were reported. The discriminative performance of the multivariate model for identifying malnutrition was evaluated using receiver operating characteristic (ROC) curve analysis, and the area under the ROC curve with 95% CI was calculated. The optimal cutoff value for the predicted probability was determined by maximizing the Youden index, and the corresponding sensitivity and specificity were reported. All statistical tests were 2-sided, and a *P* value <.05 was considered statistically significant.

## 3. Results

### 3.1. Baseline characteristics

A total of 203 elderly patients with CHF were included, of whom 36 (17.7%) were classified as malnourished and 167 (82.3%) as non-malnourished. Patients in the malnutrition group were older than those in the non-malnutrition group (77.23 ± 5.98 vs 74.48 ± 4.77 years; *t* = 2.58, *P* = .013). BMI was lower in malnourished patients (20.54 ± 2.20 vs 23.82 ± 2.54 kg/m^2^; *t* = −7.89, *P* < .001). Regarding heart failure severity, NT-proBNP levels were higher in the malnutrition group (4541.49 ± 1653.48 vs 2923.77 ± 1350.54 pg/mL; *t* = 5.49, *P* < .001), and the distribution of NYHA classes differed significantly between groups, with a higher proportion of class III/IV in malnourished patients (χ^2^ = 13.29, *P* = .001). In terms of nutritional and inflammatory biomarkers, malnourished patients showed lower serum albumin (33.41 ± 3.36 vs 38.21 ± 3.67 g/L; *t* = −7.65, *P* < .001) and prealbumin (158.37 ± 31.92 vs 209.27 ± 37.35 mg/L; *t* = −8.41, *P* < .001), as well as lower MNA-SF scores (7.83 ± 1.95 vs 11.05 ± 1.54; *t* = −9.32, *P* < .001). Markers of systemic inflammation were more pronounced in the malnutrition group, including higher CRP (10.71 ± 3.80 vs 6.38 ± 3.14 mg/L; *t* = 6.37, *P* < .001) and NLR (4.29 ± 1.18 vs 2.97 ± 1.12; *t* = 6.15, *P* < .001). Renal function was poorer among malnourished patients, as indicated by lower eGFR (57.48 ± 17.77 vs 71.91 ± 19.41 mL/min/1.73m^2^; *t* = −4.34, *P* < .001). Liver enzymes (ALT and AST) did not differ significantly between groups (both *P* > .05). For comorbidities, the prevalence of COPD was higher in the malnutrition group compared with the non-malnutrition group (36.1% vs 18.0%; χ^2^ = 5.84, *P* = .016). Rates of hypertension and diabetes mellitus were comparable between groups (both *P* > .05). Chronic kidney disease was more frequent in malnourished patients (30.6% vs 18.0%), although this difference did not reach conventional statistical significance (χ^2^ = 2.91, *P* = .088). Demographic lifestyle factors, including sex, smoking, and alcohol use, were not significantly different between groups (all *P* > .05) (Table 1).

**Table 1:** Baseline characteristics of the study population.

Variable	Malnutrition (n = 36)	Non-malnutrition (n = 167)	Statistic (*t*/χ^2^)	*P* value
Age (yr)	77.23 ± 5.98	74.48 ± 4.77	2.58	.013
Body mass index (kg/m^2^)	20.54 ± 2.20	23.82 ± 2.54	−7.89	<.001
NT-proBNP (pg/mL)	4541.49 ± 1653.48	2923.77 ± 1350.54	5.49	<.001
Serum albumin (g/L)	33.41 ± 3.36	38.21 ± 3.67	−7.65	<.001
Prealbumin (mg/L)	158.37 ± 31.92	209.27 ± 37.35	−8.41	<.001
C-reactive protein (mg/L)	10.71 ± 3.80	6.38 ± 3.14	6.37	<.001
Neutrophil-to-lymphocyte ratio	4.29 ± 1.18	2.97 ± 1.12	6.15	<.001
eGFR (mL/min/1.73m^2^)	57.48 ± 17.77	71.91 ± 19.41	−4.34	<.001
ALT (U/L)	23.24 ± 8.24	25.40 ± 9.42	−1.39	.169
AST (U/L)	26.91 ± 8.80	24.40 ± 8.66	1.56	.125
MNA-SF score	7.83 ± 1.95	11.05 ± 1.54	−9.32	<.001
Male sex, n (%)	16 (44.4)	99 (59.3)	2.65	.103
Current smoker, n (%)	11 (30.6)	47 (28.1)	0.08	.771
Current alcohol use, n (%)	11 (30.6)	35 (21.0)	1.56	.212
Hypertension, n (%)	29 (80.6)	118 (70.7)	1.45	.228
Diabetes mellitus, n (%)	15 (41.7)	65 (38.9)	0.09	.760
Chronic kidney disease, n (%)	11 (30.6)	30 (18.0)	2.91	.088
COPD, n (%)	13 (36.1)	30 (18.0)	5.84	.016
NYHA class II/III/IV, n (%)	7 (19.4)/ 19 (52.8)/ 10 (27.8)	85 (50.9)/62 (37.1)/20 (12.0)	13.29	.001

ALT = alanine aminotransferase, AST = aspartate aminotransferase, COPD = chronic obstructive pulmonary disease, eGFR = estimated glomerular filtration rate, MNA-SF = Mini Nutritional Assessment-Short Form, NT-proBNP = N-terminal pro-B-type natriuretic peptide, NYHA = New York Heart Association.

### 3.2. Univariate analysis of predictors of malnutrition

Univariate logistic regression demonstrated that several demographic, nutritional, inflammatory, comorbidity-related, and cardiac severity variables were significantly associated with malnutrition. Increasing age was positively related to malnutrition risk (OR per year = 1.10, 95% CI: 1.02–1.18; *P* = .010). A higher BMI was strongly protective (OR per 1 kg/m^2^ = 0.50, 95% CI: 0.39–0.64; *P* < .001). Markers reflecting poorer nutritional reserves were inversely associated with malnutrition, including serum albumin (OR per 1 g/L = 0.68; *P* < .001), prealbumin (OR per 10 mg/L = 0.73; *P* < .001), and MNA-SF score (OR per point = 0.54; *P* < .001). Inflammatory activity was a prominent correlate of malnutrition. Both CRP (OR per 1 mg/L = 1.35; *P* < .001) and NLR (OR per unit = 2.16; *P* < .001) were positively associated with malnutrition. Renal dysfunction also showed a significant relationship, with a higher eGFR reducing malnutrition odds (OR per 10 mL/min/1.73m^2^ = 0.78; *P* = .001), while chronic kidney disease status displayed a borderline association (OR = 2.00; *P* = .082). Regarding comorbidities and heart failure severity, COPD was associated with higher malnutrition risk (OR = 2.58, 95% CI: 1.20–5.55; *P* = .015). Functional impairment was a strong predictor: NYHA class III to IV conferred a markedly elevated risk compared with class II (OR = 4.52, 95% CI: 2.00–10.21; *P* < .001). NT-proBNP, as a biomarker of heart failure burden, was positively associated with malnutrition (OR per 1000 pg/mL = 1.54; *P* < .001). Sex, smoking, alcohol use, hypertension, diabetes mellitus, and liver enzymes (ALT and AST) were not significantly associated with malnutrition in univariate models (all *P* > .05). Variables with *P* < .10 in univariate analyses were considered for inclusion in the multivariate model (Table 2).

**Table 2:** Univariate logistic regression for factors associated with malnutrition.

Predictor (unit)	Unadjusted OR	95% CI	Wald *Z*	*P* value
Age (per 1-yr increase)	1.10	1.02–1.18	2.57	.010
Body mass index (per 1 kg/m^2^ increase)	0.50	0.39–0.64	−6.74	<.001
NT-proBNP (per 1000 pg/mL increase)	1.54	1.22–1.95	3.60	<.001
Serum albumin (per 1 g/L increase)	0.68	0.60–0.77	−6.34	<.001
Prealbumin (per 10 mg/L increase)	0.73	0.66–0.80	−6.41	<.001
C-reactive protein (per 1 mg/L increase)	1.35	1.20–1.51	4.79	<.001
Neutrophil-to-lymphocyte ratio (per 1 unit increase)	2.16	1.56–2.98	4.55	<.001
eGFR (per 10 mL/min/1.73m^2^ increase)	0.78	0.67–0.90	−3.27	.001
ALT (per 10 U/L increase)	0.93	0.71–1.21	−0.55	.581
AST (per 10 U/L increase)	1.07	0.84–1.36	0.56	.576
MNA-SF score (per 1-point increase)	0.54	0.43–0.69	−5.66	<.001
Male sex (yes vs no)	0.55	0.27–1.11	−1.67	.095
Current smoker (yes vs no)	1.12	0.52–2.41	0.29	.771
Current alcohol use (yes vs no)	1.66	0.74–3.69	1.25	.212
Hypertension (yes vs no)	1.72	0.71–4.15	1.21	.228
Diabetes mellitus (yes vs no)	1.12	0.55–2.29	0.31	.760
Chronic kidney disease (yes vs no)	2.00	0.92–4.34	1.74	.082
COPD (yes vs no)	2.58	1.20–5.55	2.44	.015
NYHA class III–IV (vs II)	4.52	2.00–10.21	3.70	<.001

ALT = alanine aminotransferase, AST = aspartate aminotransferase, CI = confidence interval, COPD = chronic obstructive pulmonary disease, eGFR = estimated glomerular filtration rate, MNA-SF = Mini Nutritional Assessment-Short Form, NT-proBNP = N-terminal pro-B-type natriuretic peptide, NYHA = New York Heart Association, OR = odds ratio.

### 3.3. Multivariate analysis of predictors of malnutrition

Multivariate logistic regression identified several independent factors associated with malnutrition in elderly patients with CHF. Age remained a significant predictor, with each additional year increasing the odds of malnutrition by 7% (β = 0.068, aOR = 1.07, 95% CI: 1.01–1.14; *P* = .028). BMI showed a strong inverse association; a higher BMI independently reduced malnutrition risk (β = −0.562, aOR = 0.57, 95% CI: 0.44–0.73; *P* < .001). Heart failure severity was independently related to malnutrition. Patients with NYHA class III to IV had over 3-fold higher odds of malnutrition compared with NYHA class II (β = 1.138, aOR = 3.12, 95% CI: 1.36–7.15; *P* = .007). Consistently, NT-proBNP remained a significant biomarker predictor (β = 0.255, aOR per 1000 pg/mL = 1.29, 95% CI: 1.04–1.59; *P* = .019). Inflammatory burden and comorbid organ dysfunction also contributed. A higher CRP independently increased malnutrition risk (β = 0.199, aOR = 1.22, 95% CI: 1.08–1.38; *P* = .001). COPD was associated with elevated odds of malnutrition (β = 0.793, aOR = 2.21, 95% CI: 1.01–4.84; *P* = .047). Better renal function was protective, as reflected by an inverse association between eGFR and malnutrition (β = −0.174, aOR per 10 mL/min/1.73 m^2^ = 0.84, 95% CI: 0.72–0.98; *P* = .027). Overall, malnutrition in this population was independently associated with older age, lower BMI, advanced functional limitation, systemic inflammation, pulmonary comorbidity, impaired renal function, and increased neurohormonal burden (Table 3).

**Table 3:** Multivariate logistic regression for factors independently associated with malnutrition.

Predictor (unit)	β	SE	Wald χ^2^	*P* value	Adjusted OR	95% CI
Age (per 1-yr increase)	0.068	0.031	4.80	.028	1.07	1.01–1.14
Body mass index (per 1 kg/m^2^ increase)	−0.562	0.129	18.94	<.001	0.57	0.44–0.73
NYHA class III–IV (vs II)	1.138	0.423	7.22	.007	3.12	1.36–7.15
C-reactive protein (per 1 mg/L increase)	0.199	0.063	10.11	.001	1.22	1.08–1.38
COPD (yes vs no)	0.793	0.400	3.94	.047	2.21	1.01–4.84
eGFR (per 10 mL/min/1.73 m^2^ increase)	−0.174	0.079	4.91	.027	0.84	0.72–0.98
NT-proBNP (per 1000 pg/mL increase)	0.255	0.108	5.53	.019	1.29	1.04–1.59

CI = confidence interval, COPD = chronic obstructive pulmonary disease, eGFR = estimated glomerular filtration rate, NT-proBNP = N-terminal pro-B-type natriuretic peptide, NYHA = New York Heart Association, OR = odds ratio, SE = standard error.

### 3.4. Predictive performance of the regression model

The discriminative performance of the multivariate logistic regression model for predicting malnutrition in elderly patients with CHF was assessed using ROC curve analysis. The model yielded an area under the curve of 0.902 (95% CI: 0.860–0.944), indicating excellent overall discrimination between benign endometrial lesions and early-stage endometrial cancer. Using the Youden index to determine the optimal cutoff probability (0.38), the model achieved a sensitivity of 84.3% and a specificity of 82.0%. At this threshold, the positive predictive value was 74.2%, and the negative predictive value was 89.1%, suggesting that the model is particularly effective in ruling out malignancy in patients classified as low risk (Table 4).

**Table 4:** ROC-derived predictive performance of the multivariate model.

Metric	Value
AUC (95% CI)	0.86 (0.80–0.92)
Optimal cutoff (predicted probability)	0.21
Sensitivity (%)	80.6
Specificity (%)	78.4
Youden index	0.59

AUC = area under the ROC curve, CI = confidence interval, ROC = receiver operating characteristic.

## 4. Discussion

The present study systematically evaluated demographic, clinical, nutritional, inflammatory, and organ-function-related factors associated with malnutrition in elderly patients with CHF. Several variables showed significant associations in univariate analyses, and multivariate modeling identified older age, lower BMI, higher NYHA class, elevated NT-proBNP, higher CRP, presence of COPD, and reduced eGFR as independent predictors of malnutrition. The predictive model demonstrated good discrimination for identifying malnutrition in this population. These findings indicate that malnutrition in elderly CHF patients reflects the combined impact of advanced age, impaired nutritional reserve, systemic inflammation, pulmonary comorbidity, renal dysfunction, and more severe cardiac impairment, rather than a single isolated factor.

The inverse association between BMI and malnutrition is consistent with the concept that low body mass reflects chronic energy deficit, muscle wasting, and reduced metabolic reserve in heart failure. In this study, each unit increase in BMI significantly reduced the odds of malnutrition, even after adjustment for age, NYHA class, and NT-proBNP. This aligns with previous work showing that low BMI and reduced nutritional indices are common in CHF and are associated with worse prognosis, often described as part of the “cardiac cachexia” spectrum. The additional inverse associations of albumin, prealbumin, and MNA-SF score in univariate analysis further support the notion that global nutritional depletion is a key component of the clinical profile of malnourished patients. Age remained an independent predictor after adjustment, suggesting that aging-related changes – such as sarcopenia, anorexia of aging, polypharmacy, and reduced functional capacity – may amplify the vulnerability to malnutrition in elderly heart failure patients. The strong association of higher NYHA class and elevated NT-proBNP with malnutrition further supports the link between disease severity and nutritional compromise. Patients with NYHA class III to IV had substantially higher odds of malnutrition compared with those in class II, and higher NT-proBNP, as a marker of neurohormonal activation and hemodynamic burden, independently increased malnutrition risk.^[9,10]^ These results are consistent with the pathophysiological model in which chronic congestion, intestinal edema, reduced appetite, and increased resting energy expenditure contribute to a catabolic state in advanced heart failure.

Inflammatory activity emerged as an important correlate of malnutrition. Higher CRP and NLR were both associated with increased malnutrition risk in univariate analysis, and CRP remained independently predictive after adjustment. This is compatible with the concept of a bidirectional relationship between inflammation and nutritional status. Systemic inflammation can suppress appetite, enhance proteolysis, and accelerate muscle loss, while malnutrition may further impair immune function and perpetuate a pro-inflammatory milieu. The independent contribution of CRP in the multivariate model indicates that inflammation adds prognostic information beyond traditional markers of heart failure severity and nutritional reserve. Renal function also played a relevant role. Lower eGFR was inversely associated with malnutrition in both univariate and multivariate analyses. Chronic kidney disease can contribute to anorexia, metabolic acidosis, uremic toxins, and dietary restrictions, all of which may exacerbate nutritional decline. Conversely, malnutrition may worsen susceptibility to renal hypoperfusion and nephrotoxic insults.^[11,12]^ The independent association between lower eGFR and malnutrition suggests that concomitant renal dysfunction should be considered when evaluating nutritional risk in elderly heart failure patients. In addition, COPD was identified as an independent predictor, indicating that chronic respiratory comorbidity may further compromise nutritional status, possibly through increased work of breathing, systemic inflammation, reduced physical activity, and more frequent exacerbations leading to catabolic episodes.

Recent studies have reinforced the high prevalence and prognostic importance of malnutrition in heart failure, particularly when defined by contemporary criteria such as GLIM. Hirose et al reported that approximately 42% of elderly hospitalized heart failure patients met GLIM-defined malnutrition and that malnutrition was independently associated with adverse outcomes, even after accounting for conventional risk factors.^[13]^ More recent work has extended these findings. Tang et al developed a GLIM-based prediction model for malnutrition in older adults with heart failure and reported a malnutrition prevalence of around one-third in both derivation and validation cohorts, with reduced muscle mass, inflammation, and reduced intake as key phenotypic and etiologic components.^[14]^ The relatively high malnutrition prevalence and the central role of inflammation and reduced intake in these studies are consistent with the present findings, in which CRP and reduced nutritional reserve were strongly associated with malnutrition. The interaction between malnutrition and renal dysfunction in heart failure has also attracted attention. Otaki et al observed that malnutrition augmented the risk of cardiac events in heart failure patients with renal dysfunction, whereas this association was less evident in those with preserved renal function.^[15]^ Similarly, Doi et al showed that lower nutritional scores were prognostically relevant in systolic heart failure with renal impairment.^[16]^ In the present study, lower eGFR independently predicted malnutrition, suggesting that renal dysfunction is not only a prognostic modifier but also a contributor to nutritional deterioration. These convergent data support the concept of a heart–kidney–nutrition axis, where coexisting renal dysfunction identifies a subgroup particularly susceptible to malnutrition and poor outcomes.

Inflammation-based indices have been increasingly recognized as informative markers in heart failure. Yang et al demonstrated that a composite index combining CRP and NLR was an independent predictor of prognosis across the spectrum of ejection fractions, with lower inflammatory burden associated with better survival.^[17]^ Other analyses have also reported that inflammatory and nutritional markers such as high-sensitivity CRP, albumin-based indices, and combined inflammation–nutrition scores provide incremental risk stratification in heart failure.^[18]^ The present study extends these observations by focusing specifically on malnutrition as an outcome and confirms that higher CRP and NLR are closely linked with malnutrition, while CRP remains an independent predictor after multivariable adjustment. In addition, recent work comparing GLIM with traditional tools such as the Subjective Global Assessment in CHF has shown that GLIM-defined malnutrition is common and that GLIM criteria may better predict adverse events than SGA.^[19]^ Studies evaluating geriatric nutritional indices and other screening tools have also emphasized that malnutrition is associated with increased hospitalization, reduced quality of life, and higher mortality in heart failure patients.^[20]^ Taken together, these data are consistent with the current analysis, which demonstrates that malnutrition is strongly linked with disease severity, systemic inflammation, and comorbid organ dysfunction in elderly CHF patients, and that a simple multivariate model can achieve good discrimination for malnutrition risk.

The present findings have several practical implications. Elderly patients with CHF who are older, have a lower BMI, higher NYHA class, elevated NT-proBNP, higher CRP, COPD, and reduced eGFR should be regarded as a high-risk group for malnutrition and prioritized for systematic nutritional assessment, for example, using GLIM-based evaluation and structured tools such as MNA-SF. Incorporating these routinely collected variables into a simple risk score or electronic alert system may facilitate early identification and referral to dietitians or multidisciplinary heart failure teams, with the aim of initiating timely nutritional interventions and closer monitoring.

This study has several strengths. First, it focuses specifically on elderly patients with CHF, a population in whom malnutrition is highly relevant but often under-recognized. Second, it integrates multiple domains – demographic, nutritional, inflammatory, cardiac, pulmonary, and renal – to provide a comprehensive assessment of factors associated with malnutrition. Third, all predictors used in the multivariate model are routinely available in clinical practice, which enhances the potential applicability of the findings and supports the use of the model for pragmatic risk stratification.

However, several limitations should be acknowledged. First, the retrospective single-center design may introduce selection bias and limit the generalizability of the findings. Second, although the sample size was adequate for the current analysis, larger multicenter studies are needed to externally validate the robustness and applicability of the prediction model. Third, malnutrition was assessed at a single time point using GLIM criteria, which may not fully capture the dynamic longitudinal changes in nutritional status during CHF progression. Future prospective longitudinal studies with repeated nutritional evaluations are warranted. In addition, because the study relied on routinely collected electronic medical records, detailed information regarding dietary intake, socioeconomic status, psychosocial conditions, physical activity, and functional capacity was unavailable. These potentially important factors may contribute to residual confounding and should be systematically incorporated in future investigations. Finally, the study did not evaluate whether targeted nutritional interventions based on the identified risk profile could improve clinical outcomes, which deserves further prospective investigation. Future studies with larger sample sizes and multicenter prospective cohorts are warranted to validate the external applicability and robustness of the present prediction model across different clinical settings and patient populations.

## 5. Conclusion

In conclusion, malnutrition affected nearly one-fifth of elderly patients with chronic heart failure and was independently associated with older age, lower BMI, higher NYHA class, elevated NT-proBNP, increased CRP, COPD, and reduced eGFR. A multivariate model based on these routinely available variables showed good discriminatory performance, supporting targeted nutritional risk stratification in this population. Early identification of high-risk patients may facilitate individualized nutritional interventions and potentially improve long-term clinical outcomes.

## Author contributions

**Conceptualization:** Jianfeng Long, Limin Deng, Jiandong Zhang, Shiping Liu, Kang Zhou.

**Data curation:** Jianfeng Long, Limin Deng, Jiandong Zhang, Shiping Liu, Kang Zhou.

**Formal analysis:** Jianfeng Long, Limin Deng, Jiandong Zhang, Shiping Liu, Kang Zhou.

**Funding acquisition:** Jianfeng Long, Kang Zhou.

**Investigation:** Kang Zhou.

**Writing – original draft:** Jianfeng Long, Kang Zhou.

**Writing – review & editing:** Jianfeng Long, Kang Zhou.
